# Associations of perioperative depression with sleep quality and physical activity levels in patients undergoing elective cardiac surgery: A prospective observational study

**DOI:** 10.1371/journal.pone.0341232

**Published:** 2026-02-10

**Authors:** Shiwei Huang, Yao Chen, Yanping Wang, Shaodan Xu, Jiayi Zhang, Tao Jiang, Xuebing Xu, Yauwai Chan, Xiaoyong Shi, Minxin Wei, Youtan Liu

**Affiliations:** 1 Department of Anesthesiology, Shenzhen Hospital, Southern Medical University, Shenzhen, China; 2 Shenzhen School of Clinical Medicine, Southern Medical University, Shenzhen, China; 3 Department of Cardiac Surgery, The University of Hong Kong-Shenzhen Hospital, Shenzhen, China; 4 Department of Anesthesiology, The University of Hong Kong-Shenzhen Hospital, Shenzhen, China; Tehran University of Medical Sciences, IRAN, ISLAMIC REPUBLIC OF

## Abstract

**Background:**

Perioperative depression is very common in patients undergoing surgery, especially major surgeries. Previous studies have shown that perioperative depression could have a negative impact on postoperative recovery and quality of life. However, few papers have focused on the depressive characteristics of patients undergoing cardiac surgeries. Therefore, the objective of this study was to prospectively investigate the incidence of depression and its associations with sleep quality and physical activity levels in patients undergoing cardiac surgeries.

**Methods:**

A total of 100 consecutive cardiac surgery patients were prospectively enrolled in the study. Perioperative depression was measured using the Patient Health Questionnaire-9 (PHQ-9). Sleep quality and physical activity levels were assessed using the Athens Insomnia Scale (AIS) questionnaire and the International Physical Activity Questionnaire-Short Form (IPQA-SF), respectively. All the data were collected and recorded preoperatively and at postoperative days 7 and 30. Independent-samples t tests and Spearman correlation analysis were used to explore the associations of depression status with sleep quality and physical activity levels. Both univariable and multivariable logistic regression were used to detect the risk factors for depression.

**Results:**

The incidence of depression increased from the preoperative level (34%, 0.27–0.46), peaked at postoperative day 7 (51%, 0.41–0.61) and slightly decreased to (47%, 0.38–0.57) at postoperative day 30. Significantly higher preoperative AIS scores were found in patients with depression than in nondepressed patients (8.00 ± 1.39 vs. 5.32 ± 1.99, *p* < 0.001). Moreover, patients with depression had significantly lower preoperative IPAQ-SF scores than did those without depression (948.32 ± 332.57 vs. 1461.65 ± 380.59, *p* < 0.001). Spearman correlation analysis indicated that preoperative depression scores were strongly correlated with AIS scores (r = 0.64, *p* < 0.001*)* and moderately correlated with IPAQ-SF scores (r = −0.44, *p* < 0.001). Risk factors for preoperative depression were age, employment status, education level, NYHA class, AIS and IPAQ-SF scores.

**Conclusions:**

Our study suggests significant associations of perioperative depression with sleep quality and physical activity levels in patients undergoing elective cardiac surgery. Patients with better sleep quality and higher levels of physical activity were significantly less likely to experience depression during the perioperative period.

**Trial registration:**

This trial was registered on November 29, 2024, in the Chinese Clinical Trial Registry (ChiCTR2400093150).

## Introduction

Depression is a prevalent and detrimental mental health condition that affects a significant proportion of the general population worldwide (i.e., more than 280 million people), but the underlying mechanism is unclear, and the current therapeutic methods are ineffective [1,2]. In 2008, the WHO ranked major depression as the third leading cause of disease burden worldwide and projected that the disease would rank first by 2030 [3]. In China, the prevalence of depression increased by 54% from 1990–2021 [4]. Surgical patients, particularly those undergoing major procedures, are at increased risk of experiencing depression [5,6]. Perioperative depression, as reported previously, can adversely impact quality of life and significantly increase the risk of postoperative complications [7]. Emerging evidence suggests that certain factors, such as female sex, younger age, living alone, and higher education levels, may serve as predictors of depression [8,9].

Surgical patients often experience poor sleep quality due to unfamiliar ward environments, noise, reduced appetite, pain, and, more commonly, significant concerns regarding their illness and impending surgery [10]. According to Gabrielle I.‘s study, depression and insomnia, one type of sleep disturbance, frequently cooccur [11], and another study reported that sleep disturbances can negatively increase pain severity [12], which has already been demonstrated as a risk factor for depression [13]. In addition, increasing evidence suggests that pain and depression share common underlying mechanisms, including similarities in neuroplasticity changes [14,15]. We therefore presume that depression is associated with sleep quality in patients undergoing elective cardiac surgery.

The physical activity levels of patients can be significantly influenced by their underlying illness and related negative emotions, which may theoretically contribute to the development of depression. However, the relationship between depression and physical activity levels remains unclear because of inconsistent findings across studies. Some research suggests that moderate exercise training can reduce both the incidence and severity of depression [16], with studies reporting an 18–25% decline in depression rates regardless of the exercise pattern [17]. In contrast, other studies have reported negative and inconclusive outcomes [18].

Despite these studies, the incidence of depression in patients undergoing elective cardiac surgery and the related factors, such as sleep quality and physical activity levels, have not been extensively and comprehensively investigated. The objectives of the present study were to evaluate the incidence of depression and identify related clinical risk factors, with a particular focus on the associations of depression with sleep quality and physical activity levels in patients undergoing elective cardiac surgery. Although traditional antidepressants remain the first-line treatment for depression, some evident limitations, such as slow onset, requirements for prolonged use, and a high rate of nonresponse, highlight the need for alternative approaches [19]. In this study, we hypothesized that better sleep quality and high levels of physical activity were associated with a lower incidence of depression during perioperative period.

## Methods

### Study design and ethics statement

A single-center prospective observational study was conducted to investigate the incidence of perioperative depression and its associations with sleep quality and physical activity levels in patients undergoing cardiac surgeries. Depression scores were collected and recorded using the Patient Health Questionnaire-9 (PHQ-9) one day before surgery and at postoperative days 7 and 30. Sleep quality and physical activity levels were assessed via the Athens Insomnia Scale (AIS) questionnaire and the International Physical Activity Questionnaire-Short Form (IPQA-SF), respectively, at the same time points. Each participant was assigned to a corresponding physician, who had received specialized training on the administration and interpretation of these various questionnaires, with no change in assessor through the three time points as per the study protocol (S1 File). The preoperative questionnaires were self-administered by patients, in a designated meeting room designed to protect patients’ privacy, the follow-up assessments at 7 and 30 days postoperatively were conducted via telephone. This prospective observational study was approved by the Ethics Committee of Hong Kong University-Shenzhen Hospital (hkuszh2024226) (S2 File) and was registered at ClinicalTrials.gov (ChiCTR2400093150). Written informed consent was obtained from all study participants. This manuscript adhered to the relevant STrengthening the Reporting of OBservational studies in Epidemiology (STROBE) guidelines (S3 File).

### Study population

We prospectively enrolled 100 consecutive patients who were scheduled to undergo elective cardiac surgery at the Hong Kong University-Shenzhen Hospital from November 1, 2024, to April 30, 2025. The inclusion criteria were as follows: 1. scheduled for elective cardiac surgery and 2. agreed to participate in the study. The exclusion criteria were as follows: 1. refused to participate in study; 2. had emergent and interventional surgery; 3. had acute and chronic heart failure with an LVEF≤40%; 4. had limited expression and communication ability; 5. were currently taking antidepressants.

### Procedures and sample size

All clinical and surgical data were collected and recorded from the Cardiovascular Surgery Department of the University of Hong Kong-Shenzhen Hospital. The baseline variables included age, sex (female), body mass index (BMI), smoking status, employment status, social security status, education level, living along status, marital status, NYHA class, left ventricular ejection fraction (LVEF%) and N-terminal pro-B-type natriuretic peptide (NT-proBNP) level. Other perioperative parameters included length of hospital stay, length of cardiac intensive care unit (CICU) stay, surgery time and total blood loss. The sample size was calculated with PASS (Version 11.0.7, NCSS LLC) software according to the following factors: 95% confidence interval, 10% margin of error and 35% expected prevalence proportion. Considering 10% failure for follow-up and assuming a two-sided α of 0.05 and a power of 80%, approximately 100 patients were needed for the study.

### Assessment of depression

The PHQ-9 is a validated tool for screening, diagnosing, monitoring, and assessing depression severity; it integrates the DSM-IV criteria with depressive symptoms [20,21]. This brief, self-administered questionnaire can be quickly scored by trained physicians. The total score ranges from 0 to 24 [22], with depression severity categorized as mild (5−9), moderate (10−14), moderately severe (15−19), or severe (20−24). A score of 10 or higher is typically used to define clinical depression [23,24]. The Chinese version of the PHQ-9, which was used in this study, has been proven to be a valid and efficient tool for screening for depression [25].

### Assessment of sleep quality

Perioperative sleep disturbance (PSD), which encompasses sleep deprivation, circadian rhythm disruption, and alterations in sleep architecture, affects 15% to 72% of patients following surgery [12,26]. Sleep quality was assessed using the AIS, with an AIS score of ≥6 indicating significant sleep disturbance, characterized by repeated nighttime interruptions or more severe disruptions [27]. The AIS consists of eight items, each rated on a 0–3 scale, with total scores ranging from 0 to 24. The outcomes were categorized as no sleep disturbance (score <4), potential sleep disturbance (score 4–6), and confirmed sleep disturbance (score >6).

### Assessment of physical activity levels

Physical activity levels were evaluated using the International Physical Activity Questionnaire-Short Form (IPAQ-SF), a tool that has been extensively validated and widely utilized in international research with strong validity [28]. The short form records the activity of four intensity levels: 1) vigorous-intensity activity such as aerobics, 2) moderate-intensity activity such as leisure cycling, 3) walking, and 4) sitting [29]. Physical inactivity was defined as a total score of less than 600 metabolic equivalent minutes per week [30].

### Statistical analysis

Statistical analysis was performed using SPSS (version 22.0. Armonk, NY: IBM Corp) and GraphPad Software (Version 9.5. GraphPad Software, Inc., La Jolla, CA, USA). Continuous variables are presented as the mean±SD or median with quartiles. Categorical variables are reported as n (%), and group comparisons were made using the chi-square test or Fisher’s exact test.

One-way repeated measures analysis of variance (ANOVA) was performed to compare the depression scores longitudinally across the assessment time points. Depression severity was divided into 2 categories on the basis of the PHQ-9 score: no depression (score of 0–9) and depression (score≥10). The rate of depression (calculated as the number of patients with depression divided by the total number of patients) and the percentage of patients with depression (calculated by multiplying the rate by 100) were calculated across assessment time points. Similarly, one-way ANOVA was performed to compare the AIS and IPAQ-SF scores longitudinally across the assessment points. Independent-samples t tests were conducted to compare the AIS and IPAQ scores preoperatively, at postoperative days 7 and 30, between patients with depression (score≥10) and those without depression(score of 0–9). The correlations of depression scores with AIS scores and IPAQ-SF scores were assessed at each corresponding time point using Spearman correlation coefficients. The strength of the correlation was interpreted as: values of r < 0.3 were considered weak, r between 0.3 and 0.5 were considered moderate, and r > 0.5 were considered strong.

Univariate logistic regression was performed to examine the associations between depression status (depression or no depression) and the following variables: age, sex (female), BMI, smoking status, employment status, social security status, education level, living along status, marital status, NYHA class, AIS score and IPAQ score preoperatively. Multivariate logistic regressions were performed, forcing all covariates with a statistical significance of <0.1 into the final model with the method of backward variable elimination. A *p* value of <0.05 was considered to indicate statistical significance.

## Results

A total of 100 patients were included in this study (Flowchart). All enrolled patients completed the three questionnaires at scheduled time points, and no instances of loss to follow-up or missing data were recorded. The mean age was 54.57 years, and 34% of the patients were female. The average BMI was 23.87 kg/m^2^. In this cohort, 51% of the patients were active smokers, and only a few patients (4%) lived alone. The other baseline characteristics of all the patients are presented in Table 1.

**Table 1:** Baseline characteristics of patients with preoperative depression compared with those of patients without preoperative depression.

	Depression (PHQ-9 ≥ 10)	No depression (PHQ-9 < 10)	*p value*
	N = 34	N = 66	
Age (years) (mean±SD)	47.09 ± 9.68	58.42 ± 10.03	<0.001
Female: n (%)	12/(35%)	22/(33%)	0.85
BMI (kg/m^2^) (mean±SD)	23.75 ± 2.48	22.93 ± 3.15	0.77
Smoking status (n):			
Yes	21	30	0.12
No	13	36	
Social security status (n):			
Yes	20	27	0.09
No	14	39	
Living alone status (n):			
Yes	2	2	0.60
No	32	64	
Employment status (n):			
Yes	29	35	<0.001
No	5	31	
College education status (n):			
Yes	14	13	0.02
No	20	53	
Marital status (n):			
Married	30	64	0.18
Single	4	2	
NYHA class:			
2	15	44	0.03
3	19	22	
LVEF (%) (median, IQR)	(0.62,0.09)	(0.62,0.06)	0.57
NT-proBNP (median, IQR)	(137.50,307.75)	(223.30,386.25)	0.32

BMI: body mass index; NYHA: New York Heart Association; LVEF: left ventricular ejection fraction; NT-proBNP: N-terminal pro-B-type natriuretic peptide.

Other parameters, such as length of hospital stay, length of stay in the CICU, surgery time, and total blood loss, were also collected and analyzed. However, there was no significant difference in these variables between patients with depression and those without depression postoperatively. All the data are presented in Table 2.

**Table 2:** Other parameters in patients with depression compared with those without depression postoperatively.

	Depression (PHQ-9 ≥ 10)	No depression (PHQ-9 < 10)		
	N = 52	N = 48	*t/t’*	*p value*
Stays of hospital(d)	23.15 ± 5.84	22.27 ± 5.93	−0.75	0.46
Stays of CICU(d)	6.71 ± 2.40	6.64 ± 3.14	−0.46	0.65
Surgery time (min)	340.85 ± 103.04	349.19 ± 83.95	0.44	0.66
Total blood loss(ml)	130.96 ± 95.82	160.42 ± 129.78	1.30	0.20

All data are presented as the mean±SD.

**Fig 1:**
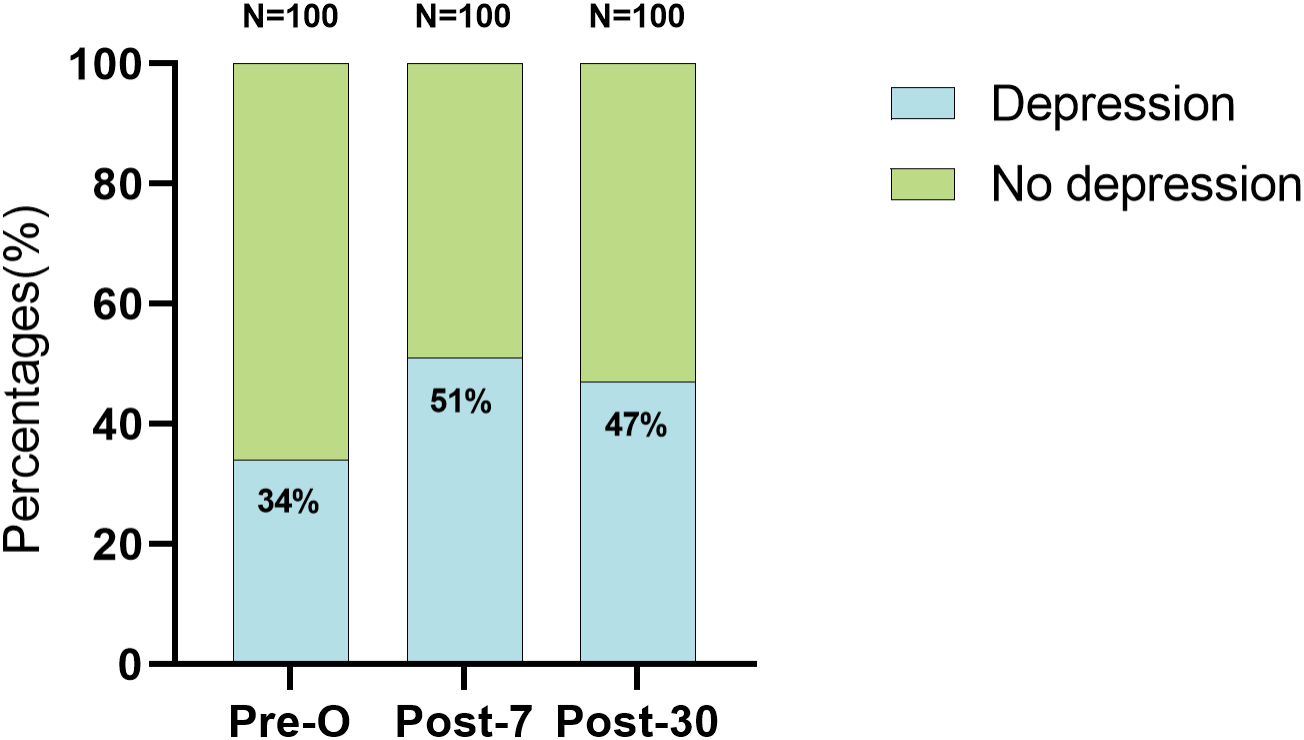
Incidence of depression in patients undergoing cardiac surgery at longitudinal time; Pre-O, Preoperative; Post-7, Postoperative day 7; Post-30, Postoperative day 30; Depression (PHQ-9 ≥ 10).

The incidence of perioperative depression increased throughout the entire study period from the preoperative level (34%, 0.27–0.46), peaked (51%, 0.41–0.61) at postoperative day 7 and slightly decreased (47%, 0.38–0.57) at postoperative day 30, remaining higher than that at baseline. This is shown in Fig 1 together with more detail.

**Fig 2:**
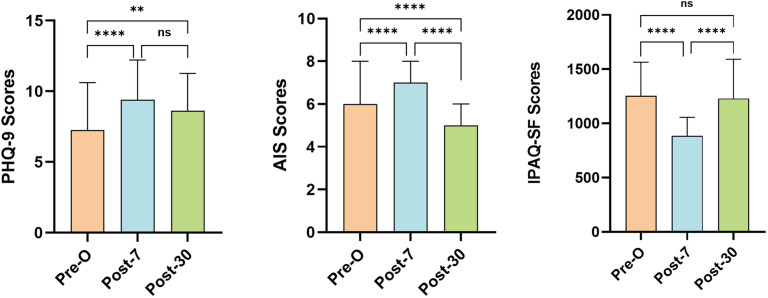
PHQ-9, AIS and IPAQ-SF scores in patients undergoing cardiac surgery at longitudinal time points; Pre-O, preoperative; Post-7, postoperative day 7; Post-30, postoperative day 30; ^****^p < 0.0001; ^**^p < 0.01; ns: not significant; Error bars represent standard deviation.

The PHQ-9 score increased significantly from baseline (7.27 ± 3.35), peaked at postoperative day 7 (9.40 ± 2.82) (*p* < 0.0001) and slightly decreased at postoperative day 30 (8.63 ± 2.63), while remaining significantly higher than that at baseline (*p =* 0.0036). On postoperative day 7, the AIS score (7.54 ± 1.53) significantly increased compared with that at baseline (6.23 ± 2.21) (*p* < 0.0001) and decreased on postoperative day 30 (4.95 ± 1.45), while remaining lower than that at baseline (*p* < 0.0001). The IPAQ-SF score was significantly lower at postoperative day 7 (879.68 ± 269.87) than at baseline (1287.12 ± 437.84) (*p* < 0.0001) and then increased at postoperative day 30 (1317.97 ± 422.52), which was significantly greater than the score at postoperative day 7 (*p* < 0.0001). No significant difference was observed between preoperative baseline (1287.12 ± 437.84) and postoperative day 30 (1317.97 ± 422.52) (*p* = 0.84) (Fig 2).

**Fig 3:**
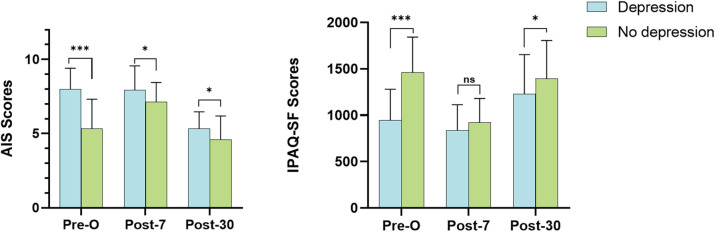
AIS and IPAQ-SF scores in patients with depression versus nondepressed patients at longitudinal time points; Pre-O, preoperative; Post-7, postoperative day 7; Post-30, postoperative day 30; ***p< 0.001; *p < 0.05; ns: not significant; Depression (PHQ-9 ≥ 10); Error bars represent standard deviation.

Significantly higher AIS scores were found in patients with depression than in patients without depression at three longitudinal time points, and the difference was greatest at the preoperative level (8.00 ± 1.39 vs. 5.32 ± 1.99, *p* < 0.001)*.* (Fig 3). The IPAQ-SF scores were significantly lower preoperatively in patients with depression than in patients without depression (948.32 ± 332.57 vs. 1461.65 ± 380.59, *p* < 0.001). At postoperative day 7, there was no significant difference in IPAQ-SF scores between the two groups (837.88 ± 276.81 vs. 923.18 ± 258.09, *p* = 0.12). Nevertheless, the difference in IPAQ-SF scores between the two groups was significant at postoperative day 30 (1230.66 ± 423.62 vs. 1395.40 ± 410.04, *p* = 0.048) (Fig 3).

**Fig 4:**
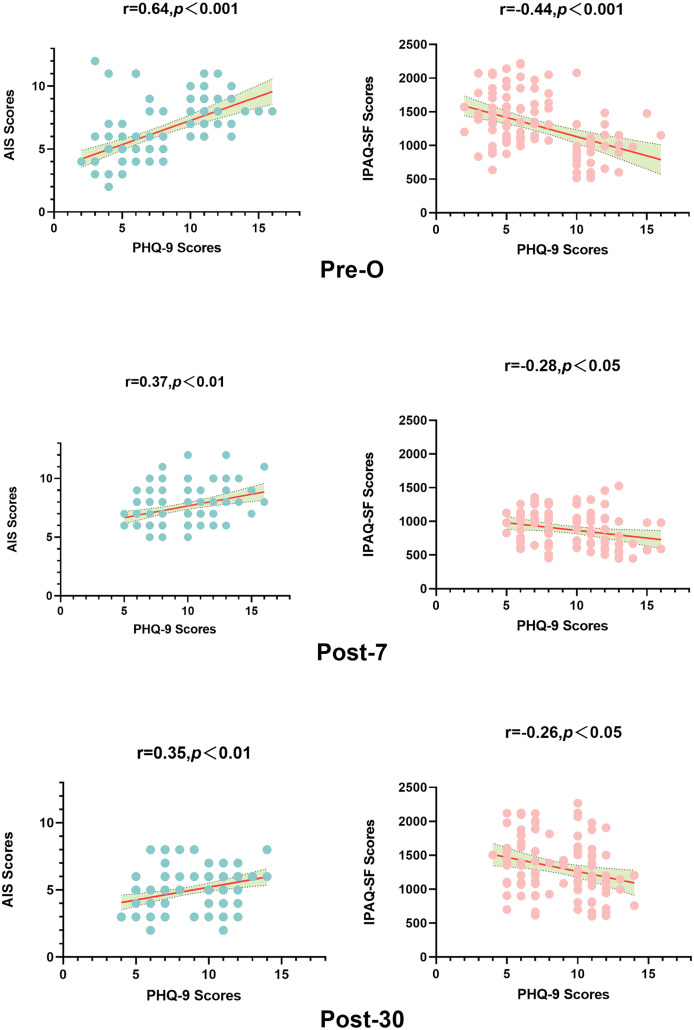
Spearman correlation analysis indicated that depression scores were significantly correlated with AIS and IPAQ-SF scores; Pre-O, preoperative; Post-7, postoperative day 7; Post-30, postoperative day 30; Red line means: 95% confidence interval.

Preoperative depression scores were strongly correlated with AIS scores (r = 0.64, *p* < 0.001*)* and moderately correlated with IPAQ-SF scores (r = −0.44, *p* < 0.001). PHQ-9 scores at postoperative days 7 and 30 were also significantly correlated with AIS scores and IPAQ-SF scores, though the intensity of the correlation was weaker. All of the correlation results are shown in Fig 4.

Univariate analysis revealed that age, employment status, education level, NHYA class, AIS scores and IPAQ-SF scores were independently associated with preoperative depression status, whereas in the multivariate regression model, which was adjusted for age, social security status, employment status, college education status, NYHA class, AIS scores and IPAQ-SF scores. Unemployment was associated with significantly reduced odds of preoperative depression (OR: 0.04, 95% CI: 0.002–0.708; P = 0.03). Conversely, the presence of sleep disturbance was associated with approximately 2.3-fold higher odds of preoperative depression (OR: 2.29, 95% CI: 1.46–3.58; P < 0.0001). Furthermore, a lower IPAQ-SF score was slightly associated with increased odds of preoperative depression (OR: 0.996, 95% CI: 0.993–0.998, P = 0.001). All of the detailed data are shown in Table 3.

**Table 3:** Univariable and multivariable analyses of independent risk factors for preoperative depression.

Variables	Univariable analysis		*p value*	Multivariable analysis		*p value*
	OR	95% CI		OR	95% CI	
Age (years)	0.9	0.85-0.94	<0.001	0.93	0.86-1.01	0.09
Female (n)	1.09	0.46-2.60	0.85	——^a^		——
BMI (kg/m^2^)	0.98	0.85-1.13	0.77	——		——
Smoking status (n):						
Yes	1.94	0.83-4.51	0.12	——		——
no	1(Ref)^b^					
Social security status (n):						
Yes	2.06	0.89-4.78	0.09	3.36	0.78-22.88	0.14
No	1(Ref)					
Living alone status (n):						
Yes	2.00	0.27-14.86	0.5	——		——
No	1(Ref)					
Employment status (n):						
Yes	1(Ref)					
No	0.20	0.07-0.57	0.003	0.04	0.002-0.708	0.03
College education status (n):						
Yes	2.85	1.15-7.12	0.02	2.13	0.26-9.23	0.40
No	1(Ref)					
Marital status (n):						
Married	4.27	0.74-24.60	0.11	——		——
Single	1(Ref)					
NYHA class						
2	1(Ref)					
3	2.53	1.08-5.92	0.03	2.41	0.46-12.67	0.30
AIS scores	2.08	1.54-2.81	<0.0001	2.29	1.46-3.58	<0.0001
IPAQ-SF scores	0.996	0.994-0.998	<0.0001	0.996	0.993-0.998	0.001

^a^: No data available ^b^: This data as constant; CI: confidence interval; OR: odds ratio.

## Discussion

The main finding of our study was that the incidence of depression among patients undergoing elective cardiac surgery was notably higher than that reported for the general population [5,6]. Preoperatively, 34% of patients exhibited depressive symptoms, which peaked at 51% at postoperative day 7 and slightly decreased to 47% at postoperative day 30. Furthermore, patients who reported better sleep quality and higher levels of physical activity had significantly lower risk of experiencing depression during the perioperative period than did those with poor sleep quality and lower levels of physical activity. These findings suggest that improving sleep quality and increasing physical activity levels may help alleviate perioperative depression.

Other clinical parameters, such as age, employment status, education level, NYHA level, AIS scores and IPAQ-SF scores, were significantly associated with preoperative depression status, which was mostly in accordance with previous studies [8,31]. Postoperative clinical outcomes, such as length of hospital stay, length of CICU stay, surgery time and total blood loss, were not different between patients who developed depression and those who did not develop depression. This finding was not the same as that of Horne, D., who reported that hospital stays longer than 7 days were associated with an increased incidence of postoperative depression [32]. Notably, depression is more prevalent in patients undergoing cardiac surgery [33,34], and its presence is associated with increased postoperative complications and severely impaired postoperative recovery and quality of life [15,35]. Preoperative depression screening [36,37] is therefore essential for identifying patients who may require additional care or treatment on the basis of the severity of their symptoms. Early intervention and treatment could reduce the risk of developing depression or mitigate its severity [38–40]. In some countries, anesthesia clinics routinely assess patients’ mental health via a variety of specialized questionnaires, recognizing its importance alongside traditional conditions [24] such as hypertension, diabetes, chronic obstructive pulmonary disease (COPD), and coronary heart disease (CHD). However, few hospitals in China use the same screening system to detect those people potentially affected by perioperative depression.

Our study confirmed a strong association between perioperative depression and poor sleep quality, which is consistent with prior research indicating that depression and sleep disturbances frequently cooccur [11,41], particularly after major surgeries [26]. Notably, approximately two-thirds of individuals with depression experience insomnia, and up to 90% report suboptimal sleep quality [42]. Preoperative depression has also been identified as a significant risk factor for postoperative sleep disturbances [27]. The underlying mechanism linking depression and poor sleep quality may involve dysregulation of the hypothalamic‒pituitary‒adrenal (HPA) axis, a common biological pathway implicated in both conditions [43].

In our cohort, patients with depression had significantly higher perioperative AIS scores than did those without depression, with the most pronounced difference observed preoperatively. We hypothesize that preoperative anxiety, fear of surgery, and the stressful hospital environment contribute to this disparity. Postoperatively, pain has been reported as a critical factor impairing sleep quality [44]. Given that poor sleep adversely affects recovery [45] and diminishes life satisfaction [46], interventions to enhance sleep quality are critical for optimizing postoperative outcomes. As prior studies have suggested [12], effective pain management may be a key strategy. Improved sleep quality could subsequently alleviate depressive symptoms and enhance overall patient well-being.

Furthermore, our study revealed significantly lower preoperative and 30-days postoperative physical activity levels in patients with depression compared with those without depression. While the intensity of the difference decreased on postoperative day 30, we attributed this change to enhanced postoperative rehabilitation training following surgery, which is a routine protocol in our hospital. Several factors may contribute to reduced physical activity levels, including the demands of the surgery, pain, and insufficient sleep. Previous studies [47–49] have suggested that moderate and appropriate exercise can have a positive effect on postoperative depression, and even daily walking can mitigate depressive symptoms and decrease the incidence of depression [50]. The optimal benefits were observed at approximately 15 hours of exercise per week [17]. According to other studies [51], exercise training has been shown to reduce depressive symptoms compared with usual care, particularly in patients requiring rehabilitation after cardiac surgery [48]. However, we did not distinguish the effects of individual forms of exercise on depression, which we believe is an interesting research topic. In addition, one study categorized patients with fewer than 600 metabolic equivalent minutes per week as inactive [30], and patients in our study meeting this criterion all experienced major depression.

Nevertheless, when interpreting the findings of our study, several potential limitations should be acknowledged. Our findings were obtained in just one center with a small sample size. Future large, multicenter studies are needed to confirm our findings. All the questionnaires used in this study were self-reported, which will introduce the potential for recall and response bias in the interpretation of the results. Additionally, when assessing sleep quality, we did not distinguish between day and night sleep. Consistent with previous reports, the degree of postoperative sleep disorders was greater in the evening than in the morning, which may be related to the circadian rhythm [52]. It should also be noted that sleep quality was only assessed subjectively, without any objective assessments such as actigraphy or polysomnography. This may introduce bias, and future studies incorporating objective assessments are warranted. Furthermore, depression was measured using a virtual self-assessment tool, the PHQ-9, as opposed to a standard diagnostic method. Finally, based on multivariable analysis, employment status, AIS scores, and IPAQ-SF scores emerged as significant risk factors associated with perioperative depression. However, our study did not collect data on other potential contributors, such as pre-existing sleep disturbances, preoperative cognitive impairment, or functional disability, which may also affect depression risk [53].

In terms of the outcomes of our study, the incidence of depression was high and was strongly associated with sleep quality and physical activity levels in patients undergoing elective cardiac surgery. These results suggest that more attention should be given perioperatively to patients undergoing elective cardiac surgery. A multimodal approach, such as physical therapy, psychological support [54], and adjunctive pharmacotherapy could provide comprehensive depression management. Although antidepressant medications remain the first-line treatment for depression in many settings, when certain notable limitations are accounted for, such as slow onset of action, the requirements for prolonged use, and high rates of nonresponse [55,56], alternative strategies warrant consideration. In contrast, sleep quality improvement and physical activity promotion are free from pharmacological side effects and are relatively easy to accomplish and generalize. These nonpharmacological interventions may be particularly valuable for patients with incomplete responses to conventional antidepressant therapy.

## Conclusions

Our study suggests that perioperative depression is significantly associated with sleep quality and physical activity levels in patients undergoing elective cardiac surgery. Notably, patients with better sleep quality and higher levels of physical activity experienced significantly lower depression during the perioperative period than did those with poor sleep quality and lower levels of physical activity. Additionally, preoperative factors such as age, employment status, education level, NYHA class, AIS scores, and IPAQ-SF scores were identified as significant predictors of depression. Given these findings, we recommend incorporating sleep quality improvement and physical activity promotion into perioperative depression management.

## Supporting information

S1 File Study protocol. (PDF)

S2 File Ethics approval. (PDF)

S3 File STROBE-checklist. (PDF)

S4 File Research date. (XLSX)
