# Supplementary material for: Associations of perioperative depression with sleep quality and physical activity levels in patients undergoing elective cardiac surgery: A prospective observational study
Source: PLoS One. 2026 Feb 10;21(2):e0341232. doi: 10.1371/journal.pone.0341232 (PMC12890135; doi:10.1371/journal.pone.0341232)
Supplement: S1 File — (PDF) [file pone.0341232.s001.pdf]

THE UNIVERSITY OF HONG KONG-SHENZHEN HOSPITAL

Scientific Research Project Ethics Review

Application Form

Project Title: Associations of perioperative depression with sleep quality and physical activity levels in patients undergoing elective cardiac surgery: A prospective observational study

Department: Department of Anesthesiology

Principal Investigator: Shiwei Huang

No.	Verification Item	Verification Result
1	Have you read the "HKU-SZH Ethics Committee Review Application Guide"?	☑Yes ☐No
2	Have you read the HKU-SZH Scientific Research Project Management Measures?	☑Yes ☐No
3	Has the PI participated in a "GCP" training course and obtained a certificate? (Required for projects involving human subjects)	☑Yes ☐No
4	Does it involve the export of human genetic resources? (If yes, please contact the Research Department for consultation and procedures)	☐Yes ☑No

一、Project Title

Associations of perioperative depression with sleep quality and physical activity levels in patients undergoing elective cardiac surgery: A prospective observational study

二、Principal Investigator Information

Name	Shiwei Huang	Phone	17790943866
Department	Anesthesiology	Email	huangsw@hku-szh.org
Main work and Research experience	Research and Academic Work Experience:<br>1. 2023-Present, Ph.D. Candidate in Anesthesiology, Southern Medical University<br>2. 2021-Present, The University of Hong Kong-Shenzhen Hospital, Department of Anesthesiology, Attending Doctor<br>3. 2017-2019, Northern Theater Command General Hospital, Department of Anesthesiology, Attending Doctor<br>4. 2014-2017 The First Affiliated Hospital of China Medical University, Department of Anesthesiology, Master of Anesthesiology<br><br>Papers<br>1. 黄世伟,王俊. 丙泊酚闭环靶控输注在中老年患者气管插管与拔管反应中的应用. 实用药物与临床. 2017;20（06）:668-671.<br>2. 黄世伟,李林. 胸椎旁神经阻滞与多点肋缘下腹横肌平面阻滞 在开腹肝脏手术术后镇痛的应用比较. 国际麻醉学与复苏杂志. 2020;41（3）:260-264.<br>3. Huang S, Wang Z, Chan Y, et al. Airway Management of an Infant With Giant Neck Macro-Cystic Hygroma Utilizing a High-Flow Nasal Cannula. Cureus. 2023.<br>4. Zhu Bowen, Gu Zheng, Huang Shiwei, Liu Youtan; Altered Gut Microbiota Contributes to Acute-Respiratory-Distress-Syndrome-Related Depression through Microglial Neuroinflammation.Research.2023		

三、Other Project Personnel Information

No.	Name	Unit and Department	Position	Role in the Project
1	Shaodan Xu	HKU-SZH, Dept. of Anesthesiology	Senior Doctor	Data Collection
2	Chen Yao	HKU-SZH, Dept. of Cardiac Surgery	Resident	Draft Manuscript Writing
3	Jiayi Zhang	HKU-SZH, Dept. of Anesthesiology	Resident	Data Collection

4	Yanping Wang	HKU-SZH, Dept. of Anesthesiology	Resident	Research Protocol Design
5	Tao Jiang	HKU-SZH, Dept. of Anesthesiology	Senior Doctor	Statistical Analysis & Software
6	Xuebing Xu	HKU-SZH, Dept. of Anesthesiology	Consultant	Draft Review & Revision
7	Xiaoyong Shi	HKU-SZH, Dept. of Anesthesiology	Consultant	Funding Support
8	Minxin Wei	HKU-SZH, Dept. of Cardiac Surgery	Consultant	Manuscript Review & Process Supervision
9	Youtan Liu	Shenzhen Hospital of Southern Medical University	Professor	Manuscript Review & Process Supervision

四、Project Research Location

Primary Research Location	The University of Hong Kong-Shenzhen Hospital
Collaborative Research Location	None

五、Project Research Timeline

Start Date	2024-11-01
End Date	2025-05-31

六、Project Research Background & Rationale （Less than 1000 words）

Research Background and Significance:

Perioperative depression, also known as major depressive disorder (MDD), is characterized by pervasive alterations in mood and cognition lasting at least two weeks, along with loss of interest or pleasure (anhedonia) in normal activities. Major depression affects over 300 million people globally, accounting for approximately 4.4% of the world's population, with an estimated lifetime prevalence of 11%. It is the single largest contributor to global disability, leading to substantially increased healthcare costs and significantly reduced quality of life. Depression is also one of the greatest risk factors for suicide. Few clinical issues are as pressing as the need to develop new treatments for depression. Moreover, depression is the leading cause of non-fatal health loss worldwide. The incidence in women is 1.5 times that in men; poverty, unemployment, life events, and physical illness all increase the risk of developing depression.

Over the past 30 years, the number of new cases globally has increased by nearly 50%, and currently more than 264 million people of all ages are affected by depression. Among them, the prevalence in the general female population ranges from 5% to 9%, and in males from 2% to 3%. However, the incidence of perioperative depression in patients scheduled for cardiac surgery is approximately 14–47%, and postoperative depression occurs in about 20–54% of cases—significantly higher than in the general population. Moreover, the estimated recurrence rate of depression is as high as 75–90%. According to the World Health Organization, depression is the leading cause of mental and physical disability worldwide and a major contributor to the global burden of disease (WHO, 2021). Even more concerning, adolescents with major depressive disorder have a suicide risk 30 times higher than non-depressed individuals.

The harms of perioperative depression include, but are not limited to: severe reduction in quality of life, such as loss of interest and enthusiasm for people and activities; increased morbidity and mortality; elevated risk of cancer; higher perioperative pain scores; disruption of sleep patterns and induction of sleep disorders; disability and suicide rates significantly higher than in the general patient population; long disease duration; high recurrence rate; and severe impairment of social and executive functioning. The Chinese Guidelines for the Prevention and Treatment of Depressive Disorders (Second Edition) note that 50–85% of patients with depression experience at least one recurrence in their lifetime.

Existing research domestically and internationally:

As reported in the literature, depression is a common comorbidity in patients with coronary artery disease, with an incidence of approximately 14–47%, often occurring in patients with unstable angina or those awaiting coronary artery bypass grafting (CABG). Additionally, about 20% of patients develop new-onset or worsened depression after CABG, which is associated with an approximately 1.4-fold increase in the risk of early and late postoperative mortality and major adverse cardiovascular events (MACE). Cardiovascular disease is the leading cause of death in the United States, making it a common target for interventional research. Therefore, the composite endpoint of "major adverse cardiovascular events" (MACE) is an increasingly common primary outcome measure, focusing mainly on acute myocardial infarction, stroke, heart failure, all-cause mortality, and revascularization procedures.

Regarding cardiac surgery research, Caspi-Avissar, N., in a study exploring predictors of depression after cardiothoracic surgery, indicated that the incidence of postoperative depression following coronary artery bypass grafting and valve replacement surgery is as high as 20–54%, far exceeding that in the general population. Drudi, L. M., in a study on the correlation between perioperative depression and mortality in elderly patients undergoing thoracoscopic or open valve replacement surgery, found that preoperative depression increased all-cause mortality at one month postoperatively by approximately threefold. In analyzing and comparing the degree of perioperative depression in patients scheduled for mitral valve surgery, Botzet, K. found that preoperative anxiety and depression were higher than in the general population, and depression levels at both early (1 week) and late (6 months) postoperative stages were elevated to varying degrees compared to baseline. Acikel, M. E. T., in an analysis of patients scheduled for coronary artery bypass grafting, found that postoperative depression levels were significantly

higher than preoperative levels; however, there was no significant difference between short-term (3 days) and long-term (30 days) postoperative assessments. Similarly, Tully, P. J., in a study analyzing depression and mortality after bypass surgery, confirmed that preoperative depression was associated with significantly increased postoperative mortality.

In summary, depression is highly prevalent among cardiac surgery patients and has serious consequences, severely reducing quality of life and increasing postoperative mortality.

七、Purpose of the Project

Perioperative depression significantly impacts patient recovery, quality of life, and overall well-being. It is reported that the incidence of perioperative depression in patients scheduled for elective surgery is approximately 12–17%. Among these, the incidence in cardiac surgery patients ranges from about 14% to 47% preoperatively, and between 20% and 54% postoperatively—far exceeding rates observed in the general surgical population. Moreover, patients with preexisting depression often experience worsening depressive symptoms after surgery, which can lead to an increased risk of major adverse cardiovascular events and even elevated mortality.

This study aims to evaluate the severity of perioperative depression in patients undergoing elective cardiac surgery at our institution using questionnaire-based assessments. The objectives include estimating the actual prevalence of depression and identifying associated risk factors in this patient population, as well as exploring the relationship between perioperative sleep quality, physical activity levels, and depression. The findings will provide evidence-based guidance on whether corresponding interventions are warranted during the perioperative period.

This constitutes the first phase of the research. If the results confirm that this patient group is indeed at high risk of depression, additional attention and treatment will be indicated. A subsequent phase will involve interventional treatment for these patients. For example, a prospective randomized controlled trial will be conducted using intraoperative administration of the novel antidepressant esketamine to evaluate its perioperative antidepressant effects. This aims to provide new evidence and options for the clinical management of depression in this setting.

八、Key Technical Metrics and Expected Outcomes of the Project

The primary observational metrics of this study are the incidence and severity of preoperative depression, assessed using the Patient Health Questionnaire-9 (PHQ-9) scoring system.

The Patient Health Questionnaire-9 (PHQ-9) is a versatile instrument used for screening, diagnosing, monitoring, and measuring the severity of depression. It incorporates the DSM-IV diagnostic criteria for depression along with other major depressive symptoms into a brief self-report tool. The PHQ-9 scores the frequency of symptoms included in the severity index, as well as the presence and duration of suicidal ideation. In addition to serving as an evidence-based auxiliary screening tool for depression, the PHQ-9 is also a reliable and valid measure of depression severity. Shulin Chen, in a study validating the reliability and validity of the PHQ-9 among primary care populations in China, confirmed that the PHQ-9 exhibits good psychometric properties and is suitable for screening elderly patients with depression in primary healthcare settings in China.

The Athens Insomnia Scale (AIS) focuses on assessing the severity of insomnia. It uses patients' subjective descriptions of symptom severity for scoring, allowing insomnia to be classified into different levels of severity. The scale has been validated in patients with primary insomnia, those with psychiatric disorders, and healthy

populations. The Chinese version of the AIS has demonstrated good psychometric properties.

The International Physical Activity Questionnaire (IPAQ) is a widely recognized and internationally used tool for measuring physical activity levels in adults (aged 15–69). It consists of two versions: short and long forms. The IPAQ has been used in studies involving Chinese populations and has been tested for good validity and reliability.

Expected Outcomes:

1、The incidence of preoperative depression among patients scheduled for elective cardiac surgery at our institution is expected to be high, comparable to previously reported rates (approximately 14–17%). This will confirm that this patient group is at high risk for depression and warrants increased clinical attention and subsequent Phase II interventional treatment.

2、An association is anticipated between perioperative sleep quality, physical activity levels, and depression: higher sleep quality and greater physical activity are expected to be inversely correlated with the incidence of depression.

九、How the Project Contributes to Medical Knowledge and Healthcare Development

1、**Evidence-Based Insights into Perioperative Mental Health:** This study aims to evaluate the incidence and risk factors of preoperative depression in patients undergoing elective cardiac surgery at our institution through standardized questionnaire surveys. It will further examine the association between perioperative sleep quality, physical activity levels, and depressive symptoms. The findings will provide high-quality evidence to support clinical decision-making regarding the need for targeted psychological and behavioral interventions during the perioperative period.

2、**Improving Clinical Outcomes through Early Identification and Intervention:** If this population is confirmed to be at high risk of depression, the results will underscore the necessity for enhanced psychological support and proactive perioperative management. Implementing tailored interventions can alleviate postoperative depressive symptoms, reduce the incidence of major adverse cardiovascular events (MACE), lower mortality, and significantly improve patients' quality of life and overall well-being.

3、**Laying the Groundwork for Novel Therapeutic Strategies:** As the first phase of a broader research initiative, this study will inform the design of a subsequent phase-II interventional trial. Should a high prevalence of depression be confirmed, a prospective randomized controlled trial will be initiated to evaluate the efficacy of intraoperative administration of es-ketamine—a novel antidepressant—in reducing perioperative depression. This has the potential to establish new evidence-based treatment options and expand clinical strategies for managing depression in surgical patients.

十、Participants

Sample size	89

Study Type: Observational study — Cross-sectional study
Sampling Method: Simple random sampling
Basis for Sample Size Calculation:
The sample size is determined based on:

1. Expected prevalence rate
2. Desired precision (margin of error)
3. Confidence level

Sample Size Calculation Formula:
$n = (Z^2 \times P \times (1 - P)) / d^2$
Where:

- Z = Z-value corresponding to the desired confidence level (e.g., 1.96 for 95% confidence)
- P = Expected prevalence rate
- d = Margin of error

Based on previous relevant literature:

- P (expected prevalence) = 0.3
- d (margin of error) = 0.05
- Confidence level = 95% (Z = 1.96)

Using a significance level (α) of 0.05 and a statistical power ($1 - \beta$) of 0.8, the sample size was calculated as n = 89 subjects via PASS software (version 11.0).
Inclusion Criteria:

1. Patients scheduled for elective cardiac surgery
2. Agreed to participate in the study

Exclusion Criteria:

1. Unwillingness to participate in the questionnaire assessment
2. Impaired communication ability that affects accurate interaction or response
3. Current use of psychotropic medications
4. Preoperative acute or chronic heart failure with EF ≤ 40%
5. Emergency or interventional procedures

十一、Benefits for Study Participants

Participants in this study will contribute valuable clinical evidence to evidence-based medicine. If the results confirm that this population is at high risk for depression, healthcare providers will be able to offer enhanced attention and active interventions to future similar patient groups. Interventions will include three main modalities: physical therapy, psychological therapy, and pharmacological treatment, tailored to the severity of depression and anxiety in each participant.

According to relevant studies, music therapy and professional psychological counseling can alleviate mild to moderate depressive symptoms, while pharmacological treatment—or medication combined with physical therapy—may be effective for moderate to severe depression. Appropriate treatment strategies will be implemented based on individual clinical presentations, thereby reducing the incidence of adverse events associated with perioperative depression and improving patients' postoperative quality of life and overall health outcomes.

十二、Participant Compensation and Compensation for Damages

The scales used in this study are internationally recognized instruments that have been extensively applied both domestically and abroad. As self-assessment tools, these scales are administered without any intervention toward the patients during completion. The results reflect objective and authentic data, and the scales themselves are not associated with causing additional psychological distress to participants. Therefore, this observational study poses no foreseeable harm to the patients.

十三、Study Risks and Protective Measures

This is an observational study that does not involve any intervention toward the patients. The study is conducted using questionnaires. Prior to participation, the purpose and procedures of the study will be explained in detail to all patients, and informed consent will be obtained.

All questionnaires used in this study are internationally recognized instruments that have been widely applied both domestically and abroad. Their validity and reliability have been well established, and they have been employed in numerous relevant studies worldwide. As self-administered assessments, these questionnaires are completed by the patients themselves without any intervention during the process. The results provide objective and authentic data, and the instruments themselves are not associated with causing additional psychological burden to participants.

Throughout the study, strict measures will be implemented to protect patient privacy. All research data will remain confidential and accessible only to the study team.

In summary, the overall risks associated with this study are considered controllable.

十四、Main Research Pathway and Methodology

Phase I of the Study (Preoperative Data Collection):

This phase involves the assessment of the prevalence and severity of depression in patients scheduled for elective cardiac surgery. The objective is to determine the actual preoperative depression levels in our patient population. If the data are consistent with previous studies—indicating that these patients exhibit significantly higher rates of depression compared to those undergoing other types of surgery—it will confirm that this group represents a high-risk population substantially affected by depression. Such findings would justify the value and necessity of implementing targeted interventions.

Main Study Procedure:

After obtaining informed consent, patients scheduled for elective cardiac surgery will be evaluated using standardized questionnaires (including, but not limited to, the PHQ-9) to assess perioperative depression, sleep quality, and physical activity levels.

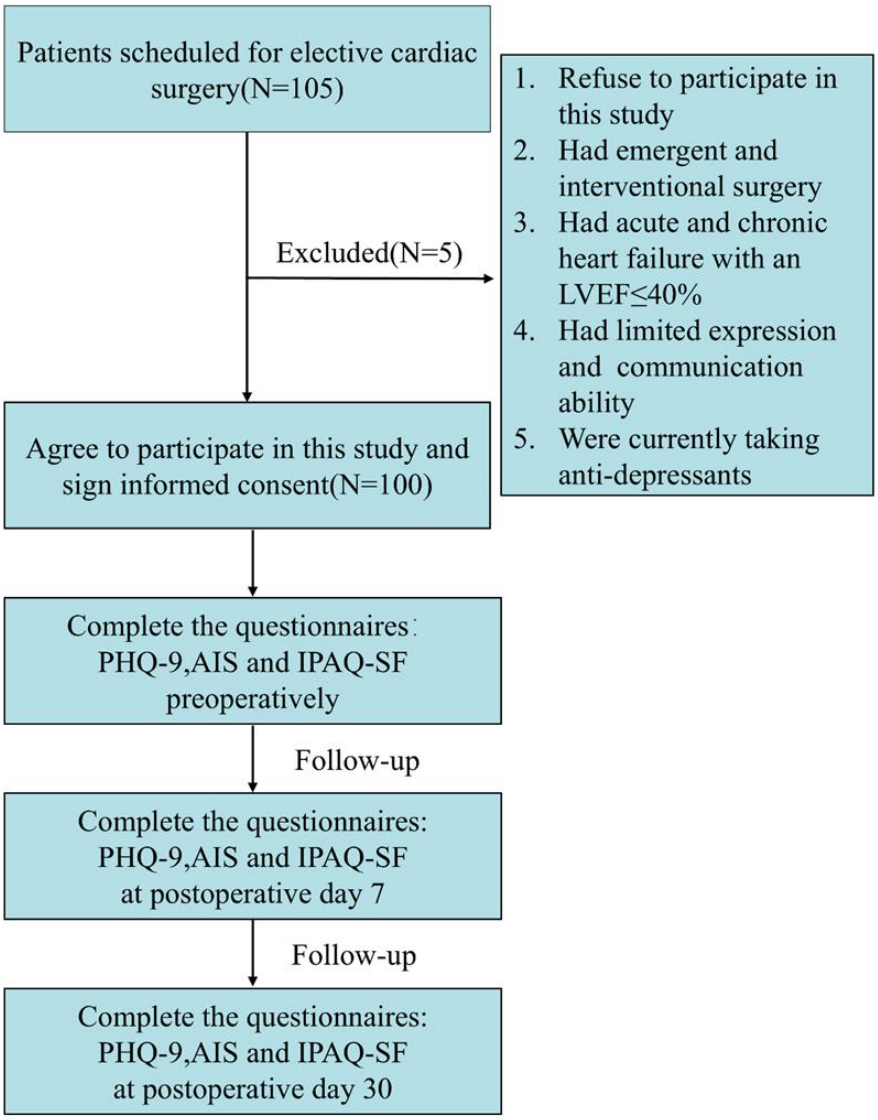

十五、Data Processing and Preservation Methods

1. Data Entry:

- Data will be recorded using both paper-based and electronic case report forms (CRFs) for synchronous preservation.
- Researchers will transcribe data from participants' original records into CRFs in a timely, accurate, complete, and legible manner.
- All data will undergo dual-entry input by two independent personnel into a designated electronic database.
- The entered data will be compared twice to ensure consistency. Any identified discrepancies will be promptly communicated to the study monitor, and the principal investigator will be required to provide clarifications.
- All data queries and resolutions will be formally documented using query forms, which shall be archived for future reference.

2. Data Verification and Management:

- After dual data entry and initial reconciliation, a data manager will perform comprehensive validation, including:
- Verification of inclusion/exclusion criteria
- Completeness checks
- Logical consistency checks
- A database validation report will be generated summarizing the quality control process and compliance with the study protocol.

3. Data Archiving:

- All completed questionnaires and CRFs will be sequentially numbered, systematically archived, and stored with a retrievable index for audit purposes.
- Electronic data files—including databases, validation scripts, analysis programs, results, codebooks, and documentation—will be categorically stored with multiple backups maintained on separate disks or storage media to prevent data loss or corruption.
- All original records will be retained in accordance with applicable regulatory requirements and institutional data retention policies.

十六、Is the hospital's existing resources sufficient to support this research?

(1) Based on previous statistics, the annual volume of coronary artery bypass grafting (CABG) and valve replacement surgeries in our hospital's cardiac surgery department averages between 200 and 250 cases. Both the surgical techniques and the entire perioperative management are well-established. A preliminary estimate indicates that the target sample size can be achieved within 4 to 5 months. We have reached a consensus with the cardiac surgery team regarding this study and have obtained their understanding and support.<br><br>(2) The research team is adequately staffed with members ranging from junior residents to consultant physicians, all of whom are qualified to fulfill the requirements of each stage of the study. This includes research design and implementation, data collection, statistical analysis, drafting of the manuscript, as well as audit and review of the paper.

No.	Required Equipment and Supporting Conditions	Description
1	Equipment Name	Not applicable
2	Department	Not applicable
3	Usage Mode	Not applicable
4	Department Head Approval Obtained☑ Yes ☐ No	

十八、Key Ethical Considerations of the Study

All questionnaire-based information provided by study participants will be treated with strict confidentiality. Access to this data will be restricted to members of the research team only, and no identifying information will be disclosed to any third party.<br><br>Throughout the questionnaire administration process, particular attention will be paid to the manner and attitude of questioning to ensure respect for participants' autonomy, preferences, and privacy. All interactions will be conducted in a professional and sensitive manner to minimize any potential discomfort and to uphold the dignity of the participants.

十九、Type of Ethics Review

(Based on the Clinical Research Classification Criteria)

Full Board Review ☐ Expedited Review ☑

二十、Commitment by the Principal Investigator

I hereby affirm that all information and materials submitted for this study are truthful, accurate, and valid, and are consistent with the documents provided to the Academic Committee.

I confirm that this research complies with all applicable ethical principles.

I commit to ensuring that the study adheres to all relevant institutional policies and will follow the requirements of the Hospital Ethics Committee, accepting its ongoing supervision and review.

I declare that this research involves no actions that violate standards of scientific integrity, including but not limited to:

(1) Plagiarism, misappropriation, or unauthorized use of others' research results;
(2) Fabrication of the research process, or falsification or manipulation of research data, figures, or conclusions;
(3) Purchasing or commissioning others to write research papers or grant applications, or inventing peer reviewers or review comments;
(4) Obtaining research projects, funding, awards, honors, or professional positions through false pretenses, bribery, exchange of benefits, or other improper means;
(5) Violation of ethical norms related to human life and health, or laboratory animal welfare;
(6) Breach of regulations regarding authorship credit or publication ethics;
(7) Any other acts of academic misconduct.

In the event of any violation, I accept all disciplinary actions imposed by the project management authority and other relevant bodies.

Signature of Principal Investigator：Shiwei Huang

二十一、Comments from Department Head （or Authorized Representative）

The project has been reviewed and is acknowledged to:
☑ Possess scientific merit
☑ Comply with medical ethical principles
Review Decision:
☑ Approved to proceed
Additional Comments (if any):

Department Head （or Authorized Representative） Signature：Xuebing Xu

香 港 大 学 深 圳 医 院

科研项目伦理审查

申 请 表

项目名称：择期心脏手术患者围术期抑郁与睡眠质量及体力活动水平的相关性：一项前瞻性观察研究

申请科室：麻醉医学部

项目负责人：黄世伟

序号	核查事项	核查结果
1	是否有阅读"香港大学深圳医院伦理委员会审查申请指南"	☑是 □否
2	是否阅读香港大学深圳医院科研项目管理办法	☑是 □否
3	PI 是否有参加"GCP"培训课程，取得培训证书（若为涉及人的项目，必须提供）	☑是 □否
4	是否涉及人类遗传资源出境（若有，请联系科研部咨询办理）	□是 ☑否

一、项目名称

**择期心脏手术患者围术期抑郁与睡眠质量及体力活动水平的相关性：一项前瞻性观察研究**

二、项目负责人信息

姓名	黄世伟	电话	17790943866
部门	麻醉医学部	邮箱	huangsw@hku-szh.org
主要工作及研究经历	科研与学术工作经历：<br>1.2023-至今，南方医科大学麻醉学博士在读<br>2.2021- 至今，香港大学深圳医院，麻醉科，主治医师<br>3.2017-2019，北部战区总医院，麻醉科，主治医师<br>4.2014-2017 中国医科大学附属第一医院，麻醉科，麻醉学硕士<br><br>期刊论文<br>1. 黄世伟,王俊. 丙泊酚闭环靶控输注在中老年患者气管插管与拔管反应中的应用. 实用药物与临床. 2017;20（06）:668-671.<br>2. 黄世伟,李林. 胸椎旁神经阻滞与多点肋缘下腹横肌平面阻滞 在开腹肝脏手术术后镇痛的应用比较. 国际麻醉学与复苏杂志. 2020;41（3）:260-264.<br>3. Huang S, Wang Z, Chan Y, et al. Airway Management of an Infant With Giant Neck Macro-Cystic Hygroma Utilizing a High-Flow Nasal Cannula. Cureus. 2023.<br>4. Zhu Bowen, Gu Zheng, Huang Shiwei, Liu Youtan; Altered Gut Microbiota Contributes to Acute-Respiratory-Distress-Syndrome-Related Depression through Microglial Neuroinflammation.Research.2023		

三、其他项目人员信息

序号	姓名	单位和部门	职务	在项目中所起的作用
1	许少丹	香港大学深圳医院麻醉医学部	高级医生	数据收集
2	陈瑶	香港大学深圳医院心脏外科	驻院医生	初稿撰写
3	张佳一	香港大学深圳医院麻醉医学部	驻院医生	数据收集
4	王艳娉	香港大学深圳医院麻醉医学部	驻院医生	研究方案设计

5	姜涛	香港大学深圳医院麻醉医学部	高级医生	统计分析与软件操作
6	许学兵	香港大学深圳医院麻醉医学部	顾问医生	初稿审阅与修订
7	史晓勇	香港大学深圳医院麻醉医学部	顾问医生	基金支持
8	魏民新	香港大学深圳医院心脏外科	顾问医生	论文审阅与过程监督
9	刘友坦	南方医科大深圳医院	教授	论文审阅与过程监督

四、项目研究地点

主要研究地点	香港大学深圳医院
合作研究地点	无

五、项目研究时间表

起始时间	2024-11-01
结束时间	2025-05-31

六、项目研究背景、依据（小于 1000 字）

研究背景及意义：

围术期抑郁症，也被称为临床抑郁症，其特征是至少 2 周的情绪和认知的普遍变化，以及对正常活动失去兴趣或乐趣（快感缺乏）。重度抑郁症影响全球超过 3 亿人，约占全球总人口 4.4%，估计终生患病率为 11%。它是全球残疾的最大单一因素，造成医疗成本大量增加和生活质量严重下降。抑郁症也是自杀的最大危险因素之一。很少有临床问题像需要开发新的抑郁症治疗方法一样迫切。此外，抑郁症也是全球非致命性健康损失的最大单一因素。女性的发病率是男性的 1.5 倍；贫穷、失业、生活事件和疾病都会增加患抑郁症的风险。

据报道，在过去 30 年里，全球新发病例数量增加了近 50%，目前有 2.64 亿多各年龄段的人正在受到抑郁的影响。其中，女性一般人群的患病率为 5%～9%，男性为 2%～3%。而拟行心脏手术患者围术期抑郁发生率约为 14-47%，术后抑郁发生率约为 20-54%，这一比例远高于一般人群。且抑郁复发率估计高达 75% - 90%。根据世界卫生组织的数据，抑郁症是全球精神和身体残疾的主要原因，是全球疾病负担的主要贡献者 (WHO2021)。更令人担忧的是，患有严重抑郁障碍的青少年自杀的倾向是非抑郁患者的 30 倍。

围术期抑郁的危害包括但不限于：严重降低患者生活质量，如对周围人和事情失去兴趣和热情、增加发病率和死亡率、增加患癌症的几率、增加围手术期的疼痛评分、扰乱睡眠程序、引发睡眠障碍、致残率、自杀率显著高于普通患者，且病程周期长、复发率高、社会功能与执行功能严重受损-《中国抑郁障碍防治指南（第

二版）》提到，50%-85%的抑郁症患者在其一生中至少经历 1 次复发。

国内外已有研究：

据文献所报道，抑郁症是冠状动脉疾病患者的常见合并症，发生率约为 14% -47%，多发生于不稳定型心绞痛或等待冠状动脉旁路移植术（CABG）的患者。同时约有 20%的人接受 CABG 后新发抑郁或抑郁加重，导致术后早期和晚期死亡率及重大不良心血管事件（Major Adverse Cardiovascular Event，MACE）的风险增加相关的风险增加约 1.4 倍。心血管疾病是美国的主要死亡原因，使其成为介入研究的共同目标。因此，“主要不良心血管事件”（MACE）的复合终点是一个越来越常见的主要研究结果。其主要关注指标包括：急性心梗、卒中、心衰、全因死亡率及血管再通手术等。

关于心脏手术方面的研究，Caspi-Avissar, N.在探索心胸术后抑郁发生的预测因素一文中表明，冠状动脉旁路移植术和瓣膜置换术中，术后抑郁发生率高达 20% - 54%，远高于一般人群。Drudi, L. M.在研究老年患者行胸腔镜或开胸瓣膜置换术，围术期抑郁与死亡率相关性一文中得到结果，术前抑郁会增加此类患者术后 1 个月的全因死亡率，约为 3 倍。在分析比较拟行二尖瓣手术患者的围术期抑郁程度时，Botzet, K.发现，此类患者术前焦虑和抑郁均较普通人群高，术后早期（1 周）和晚期（6 个月）的抑郁程度较基础值也有不同程度的升高。Acikel, M. E. T.在对拟行冠脉旁路移植术的患者进行分析时发现，此类患者术后抑郁程度较术前明显增加；但术后短期（3 天）和长期（30 天）比较，并无明显差异。同时，Tully, P. J.在分析抑郁与搭桥术后死亡率的文章中也证实，术前存在抑郁的患者，术后死亡率显著增加。

综上所述，抑郁在心脏手术患者中发病率高，且后果严重，严重降低患者生活质量，增加术后死亡率。

七、项目研究目的

围术期抑郁严重影响患者术后恢复情况、生活质量和健康福祉。据报道，拟行择期手术的患者围术期抑郁发生率约为 12-17%。其中心脏手术患者围术期抑郁发生率约为 14-47%，术后抑郁发生率约为 20-54%，这一比例远高于一般患者。且术前存在抑郁的患者术后抑郁程度往往会加重，这会导致此类患者术后重大不良心血管事件的发生，甚至死亡率的增加。

本研究拟对在本院行择期心脏手术的患者，以调查问卷的形式，进行围术期抑郁程度评估。估算本院此类患者实际抑郁的发生率和相关危险因素，以及探索围术期睡眠质量和运动量与抑郁的关联，为围术期是否需要进行相对应的干预治疗提供循证医学证据。

本次研究为研究**第一阶段**，获得最终结果后，若证实此类人群确为抑郁高危人群，需要给与额外的关注和治疗。后续会对此类患者进行**第二阶段的干预治疗**。如术中使用新型抗抑郁药物：艾司氯胺酮，进行前瞻性随机化分组干预治疗，探索艾司氯胺酮围术期抗抑郁的作用，为临床上治疗抑郁症提供新的依据和选择。

八、项目主要实现的技术指标及期望的结果

本研究主要的观察指标为术前抑郁的发生比例和严重程度，采用的评分标准为 PHQ-9 评分量表。

病人健康问卷-9（Patients Health Questionnaire-9），PHQ-9 是一种用于筛查、诊断、监测和测量抑郁症严重程度的多功能工具，PHQ-9 将 DSM-IV 抑郁诊断标准和其他主要的严重抑郁症状纳入一个简短的自我报告工具，该工具对纳入评分严重指数症状的频率进行评分，以及自杀意念的存在和持续时间。除了对抑郁症基于一定标准的辅助筛查外，PHQ-9 也是一种可靠的具有一定效力的抑郁严重程度的测量方法。Shulin Chen 在验证 PHQ-9 在中国基层医疗机构人群中的可靠性和有效性一文中也证实，PHQ-9 具有良好的心理测量学特性，可用于中国基层医疗机构的老年抑郁症患者的筛查。

阿 森 斯 失 眠 量 表 （Athens Insomnia Scale，AIS） 侧重评估失眠的严重程度，通过患者对于严重程度的主观描述计分，可将失眠划分为不同程度。该量表在原发性失眠患者、精神疾病患者和健康人群中被证明是有效的。中文版 AIS 具有良好的心理测量学特性。

国际体力活动量表（International Physical Activity Questionnaire, IPAQ）：是目前公认有效、 且在国际上较为广泛使用的成年人（15～69 岁）体 力活动水平测量问卷之一，分为短卷和长卷 2 个版本，已用于中国人群研究，经检验具有较好的效度与信度。

预期结果：
1、本院拟行择期心脏手术的患者术前抑郁发生率高，与以往研究报道可比，约为 14-17%。证实此类患者为抑郁高危人群，需要得到更多的关注以及后续第二阶段的干预治疗。
2、围术期睡眠质量和运动量与抑郁存在关联：睡眠质量和运动量的高低与抑郁发生率存在反向关联；

九、项目研究如何对医学知识及医疗发展做出贡献

1、本研究拟对在本院行择期心脏手术的患者，以调查问卷的形式，探索此类患者实际术前抑郁的发生率和相关危险因素，以及探索围术期睡眠质量和运动量与抑郁的关联，为围术期是否需要进行相对应的干预治疗提供循证医学证据。

2、若证实此类人群为抑郁高危人群，需要更多的心理关注和积极有效的围术期治疗措施，从而降低此类患者术后抑郁严重程度，减少严重不良心血管事件发生率，提高生存率，提高生活质量和健康福祉。

3、本次研究为研究第一阶段，获得最终结果后，若证实此类人群确为抑郁高危人群，后续会对此类患者进行第二阶段的干预治疗。如术中使用新型抗抑郁药物：艾司氯胺酮，进行前瞻性随机化分组干预治疗，探索艾司氯胺酮围术期抗抑郁的作用，为临床上治疗抑郁症提供新的依据和选择。

十、研究参与者

样本量	89 例

研究类型：观察性研究-横断面研究
抽样方法：单纯随机抽样
样本量计算依据：1、预期患病率 2、调查的精度（误差幅度）3、置信程度

样本量计算公式：$n = (Z^2 \times P \times (1-P)) / d^2$；Z=置信度、P=预期患病率、d=精读（误差幅度）
根据以往相关文献报道：本研究 P 取值为 0.3，相应精度 d 取值为 0.05, 置信度 Z 取值为：0.95
检验水准 α 取值 0.05，检验效能 1-β 取值 0.8；由 PASS 软件（11.0 版本）计算样本量计算为 n=89 例

纳入标准：
1.拟行择期心脏手术的患者
2.同意参加此项研究试验
排除标准：
1. 拒绝参加此次调查问卷评估
2. 表达能力受限，无法正确交流和回答问题
3. 正在服用抗精神疾病药物的人群
4. 术前急慢性心衰，EF≤40%
5. 急诊手术和介入手术

十一、研究参与者在研究中的获益

参与研究者为询证医学提供了宝贵的临床证据，若调查结果证实此类人群为抑郁高危人群，后续对同类型人群医务工作者需给予格外关注和积极有效的干预治疗，治疗包括三个方面：物理治疗、心理治疗和药物治疗，根据受试者抑郁和焦虑程度采取不同的干预方式。据相关报道，音乐和专业的心理疏导可以缓解轻-中度抑郁症状，药物治疗甚至药物结合物理治疗可以缓解中-重度抑郁症状。应根据具体的情况采取相应的治疗措施，从而降低围术期抑郁导致的不良事件，改善患者术后生活质量和健康福祉。

十二、研究参与者补偿及损害赔偿

本研究所采用的量表均为国际通用量表，已经在国内外得到广泛的应用。且量表为患者自评量表，在填写过程中不会对患者进行任何干预，量表结果反应的是客观真实的数据，不存在因为量表本身给患者带来额外的心理压力。所以此项观察研究不会对患者造成损害。

十三、研究风险及保护措施

此项研究为观察性研究，不对患者进行干预。研究形式为调查问卷，研究开始前会详细向患者阐述研究目的及研究过程，并取得患者知情同意。研究中所有调查问卷均为国际通用问卷，已在国内外得到广泛应用，其

效度和可信度也得到很好的验证，很多国内外相关文献都采用的研究中所使用的问卷。问卷本身为患者自评自述，此过程中不会对患者进行任何干预，问卷结果为客观真实数据。更不存在量表本身给患者带来额外心理负担的可能。研究过程中会全程注意保护患者隐私，研究数据仅限于研究团队内部知晓。
综上所述，本研究总体风险可控。

十四、主要研究路线及方法（请附上研究路线图）

本次调查为研究第一阶段（术前信息收集阶段）： 通过对拟行择期心脏手术的患者进行抑郁发生比例和严重程度的评估，确定本院患者真实术前抑郁程度，如相关数据同以往研究报道可比，即此类患者术前抑郁程度显著高于其它类型手术患者，则代表此类人群确为受抑郁影响较为严重的潜在人群，有拟行干预治疗的价值和必要性。

主要研究路线：对拟行择期心脏手术的患者，在取得知情同意后，以调查问卷（PHQ-9 等）形式，对患者围术期抑郁、睡眠质量以及体力活动量进行评估。

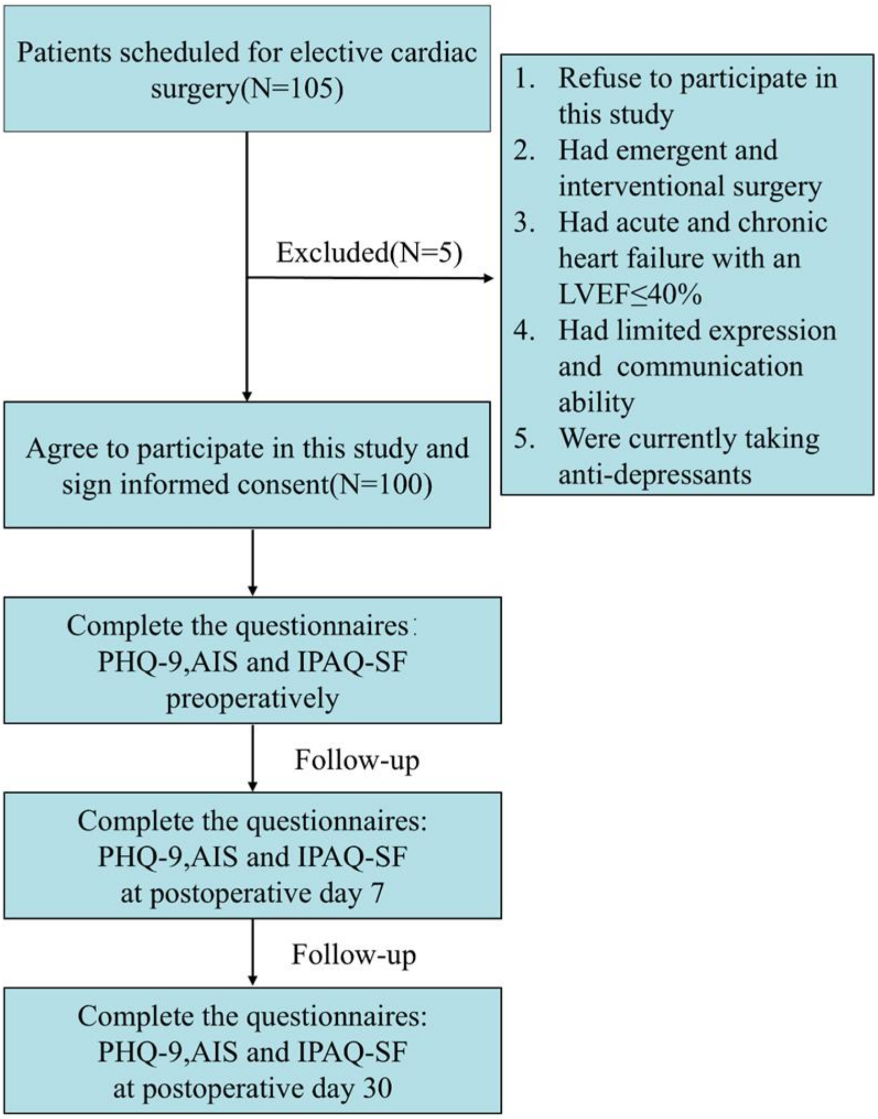

十五、数据处理方法及数据的保存

数据录入

数据记录将选择纸质/电子记录表格两种录入方式同步保存。研究者根据受试者的原始观察记录，将数据及时、完整、正确、清晰地载入病例报告表。经过双人核实后，进行数据分析比对。

录入将采用对相应数据进行双人录入，之后对数据库进行两遍比对，期间若发现问题及时通知监查员，要求研究者做出回答。他们之间的各种疑问及解答的交换应当采用疑问表形式，疑问表应保存备查。

数据核查和管理的内容和方式

所有调查问卷表经双份输入并核对无误后，由数据管理员写出数据库检查报告，其内容包括研究完成情况：入选/排除标准检查、完整性检查、逻辑一致性检查等。

数据存档

调查问卷表在按要求完成数据录入和核查后，按编号的顺序归档保存，并填有检索目录等，以备查考。电子数据文件包括数据库、检查程序、分析程序、分析结果、编码本和说明文件等，应分类保存，并有多个备份保存于不同磁盘或记录介质上，妥善保存，防止损坏。所有原始档案应按相应规定内的期限保存。

十六、医院现有资源是否支持该研究

(1)据以往统计，我院心脏外科每年平均搭桥手术和瓣膜置换术总量在 200-250 例左右，从手术技术到整个围术期管理流程都十分成熟。初步估算，可以满足在 4-5 个月内间完成样本量收集工作。已经和心脏外科团队就本研究达成共识，取得心脏外科团队的理解和支持。

(2)本研究团队人员充足，人员资质从年轻住院医生到顾问医生，可以胜任研究各个阶段的工作需求，包括：研究设计和执行、数据收集、统计学分析、论文初稿写作、论文审计和检阅等。

序号	所需设备	支持条件
1	设备名称	无需设备
2	设备所在科室	无需设备
3	设备使用方式	无需设备
4	是否取得部门主管同意： ☑ 是 ☐ 否	

十八、研究主要伦理问题

本研究会对受试者提供的调查问卷信息进行严格的保密处理，除本研究团队成员外，不会对任何人泄露上述信息。在调查问卷过程中，会注重询问的方式方法和询问态度，尊重患者的意愿和隐私。

十九、伦理审查类型（请根据“临床研究分类表”做初步判断）

会议审查 □　　　　　　简易审查 ☑

二十、项目负责人承诺

1、我承诺该研究的申报材料和相关内容真实、准确、有效，并与递交至学术委员会的材料内容一致；
2、我承诺该研究符合相关伦理原则；
3、我承诺该研究符合医院的相关政策，并遵循医院伦理委员会要求，接受伦理的持续监督与审查；
4、我承诺该研究不存在下列违背科研诚信要求的行为（包含但不限于）：
（1）抄袭、剽窃、侵占他人研究成果；
（2）编制研究过程，伪造、篡改研究数据、图表、结论；
（3）购买、代写论文或项目申请书，虚构同行评议专家及评议意见；
（4）以故意提供虚假信息等弄虚作假的方式或采取贿赂、利益交换等不正当手段获取科技计划项目、科研经费、奖励、荣誉、职务职称等；
（5）违反涉及人类生命健康、实验动物保护等科研伦理规范；
（6）违反研究成果署名、论文发表规范；
（7）其他科研失信行为。
如有违反，本人愿接受项目管理机构和相关部门做出的各项处理决定。

项目负责人签字：黄世伟

二十一、科室主管（或授权人）意见

已审查项目，并认同项目：

☑ 具有科学性

☑ 符合医学伦理原则

具体意见为：

☑ 同意项目进行

其它意见（若有）

科室主管（或授权人）签字：许学兵
