# Supplementary material for: Associations of perioperative depression with sleep quality and physical activity levels in patients undergoing elective cardiac surgery: A prospective observational study
Source: PLoS One. 2026 Feb 10;21(2):e0341232. doi: 10.1371/journal.pone.0341232 (PMC12890135; doi:10.1371/journal.pone.0341232)
Supplement: S2 File — (PDF) [file pone.0341232.s002.pdf]

The University of Hong Kong-Shenzhen Hospital Medical Ethics Committee (HKU-SZH Ethics Committee)

Review Decision Letter

Room 1306, Science and Education Building, The University of Hong Kong-Shenzhen Hospital, Haiyuan 1st Road, Futian District, Shenzhen

Tel: (0755) 86913175 Fax: (0755) 86913191

The HKU-SZH Ethics Committee is an independent committee established by HKU-Shenzhen Hospital, adhering to ICH-GCP, GCP, national relevant regulations, and other applicable standards. It performs the duties of ethical and scientific review and supervision of medical research projects according to the Committee's Standard Operating Procedures.

Date: 2024.10.25 Approval No.: Lun [2024]337

To: Huang Shiwei

Resident Doctor

Department of Anesthesiology

This letter constitutes the review decision issued by this Ethics Committee regarding the application/submission you submitted as the Investigator for the following clinical research:

- **Project Title:** Investigation of the Severity of Preoperative Depression and Anxiety in Patients Undergoing Elective Cardiac Surgery—An Observational Study
- **Project Number:** hkuszh2024226

- **Research Center:** The University of Hong Kong-Shenzhen Hospital
- **Coordinating Investigator (if applicable):** (If a multicenter clinical study and the Investigator and Coordinating Investigator are not the same person)

This Committee has conducted adequate ethical and scientific review of your application/submission according to the Standard Operating Procedures, details as follows:

- **Application/Submission Category:** ☑ Initial Review □ Re-review

  □ Periodic Follow-up Review □ Amendment Review

  □ Conclusion Review □ New Information Review

  □ Serious Adverse Event Review □ Non-compliance/Protocol Deviation Review

  □ Other
- **Review Method:** □ Meeting Review ☑ Expedited Review
- **Review Date:** 2024.10.23
- **Review Committee/Member Details:**

  Committee Name: Scientific Research Project Ethics Review Committee

  - Voting Details:

    Total Committee Members: 33 persons, Voting Members: 2 persons.

    - Votes for Approval: 2; Votes for Approval after Modifications (Expedited Review): 0; Votes for Approval after Modifications (Meeting Review): 0;
    - Votes for Disapproval: 0; Abstentions: 0;

        - Total Votes: 2.

- **Reviewed Documents:** See Appendix I for details
- **Reviewing Members:** See Appendix II for details

After review by this Committee's Review Board, this Committee has made a decision regarding your application / submission, which is notified as follows:

- **Review Decision:** Approved
- **Comments (if applicable):**
- **Periodic Follow-up Review:** During the clinical research period and counting from the initial review approval date, submit a periodic follow-up review report every (□3 □6 ☑ 12 □ Not Applicable) months.

As the Investigator of this medical research, you must conduct the research according to the requirements of this Ethics Committee, maintain close communication with this Committee during the research period, and undertake the responsibilities of the Investigator, including (but not limited to):

- Note and comply with all applicable requirements within this Committee's Standard Operating Procedures;
- Submit periodic follow-up reports according to the timelines required by this Committee's Standard Operating Procedures and this approval document;
- Do not implement any modifications/changes to the research protocol or study documents/materials without prior written approval from this Committee, unless necessary to eliminate any immediate hazard(s) to the subjects, or the modifications/changes are solely of an administrative or logistical nature;

- Report to this Committee any new information that may adversely affect the rights, safety, and health of subjects or the proper conduct of the clinical research;
- Report to this Committee any non-compliance/protocol deviation events that occur during the research period and may adversely affect the rights, safety, and health of subjects;
- Submit reports of Serious Adverse Events found within your research center and Suspected Unexpected Serious Adverse Reactions (SUSARs) reported from outside the research center, according to the requirements of this Committee's Standard Operating Procedures;
- Submit a Suspension/Termination of Clinical Research Review Application Form before pausing or terminating the research, and submit a conclusion report after study completion, according to the requirements of this Committee's Standard Operating Procedures.

Poon Waisang

Vice Chairperson

The University of Hong Kong-Shenzhen Hospital Scientific Research Project Ethics Review Committee

Consultant Doctor, HKU-SZH Neural Medicine Centre

Seal of The University of Hong Kong-Shenzhen Hospital Medical Ethics Committee

Appendix I:

Reviewed Documents

Documents reviewed by the HKU-Shenzhen Hospital Medical Ethics Committee include:

1. Initial Review Application.
2. Application Form.
3. Project Materials Integrity Commitment Letter.
4. Research Protocol (Version No.: V2.0, Version Date: 2024.09.30).
5. Informed Consent Form (Version No.: V1.0, Version Date: 2024.10.18).
6. Scientific Review Opinion.
7. Conflict of Interest Declaration.
8. List of Research Staff.
9. Questionnaire Form (Chinese and English versions).
10. References.

Appendix II:

List of Review Committee Members

Members who participated in the review of the relevant application/submission and made the review decision on behalf of the HKU-Shenzhen Hospital Medical Ethics Committee include:

Name	Profession & Affiliation	Gender	Classifications			
			Scientific Member	Non-Scientific Member	Legal Expert Member	Independent Member
Poon Waisang	Consultant Doctor; Neural Medicine Centre; HKU-SZH	Male	√			
Chen Peiling	Deputy Head, Department of Pharmacy, HKU-SZH	Female	√			

香港大学　　　　香港大学深圳医院

香港大学深圳医院医学伦理委员会
（港大深圳医院伦理委员会）
审查决定函

深圳市福田区海园一路香港大学深圳医院科教楼 1306
Tel:（0755）86913175　　Fax:（0755）86913191

港大深圳医院伦理委员会乃港大深圳医院成立之独立委员会，遵循 ICH-GCP、GCP 以及国家相关规范及其他适用标准，根据伦理委员会的标准操作规程执行对医学研究项目的伦理与科学审查和监督的职责。

日期：　2024.10.25　　　　批件号：　伦[2024]337

送交：　黄世伟
驻院医生
麻醉医学部

本函是本伦理委员会就您作为下列的临床研究之研究者而提交的申请 / 呈递而发出之审查决定：

- 项目名称：　择期心脏手术患者术前抑郁和焦虑严重程度调查--一项观察性研究
- 项目编号 ：　hkuszh2024226
- 研究中心：　香港大学深圳医院
- 协调研究者（如适用）：　（如为多中心临床研究而研究者及协调研究者并非同一人）

本会已按标准操作规程对 您的申请 / 呈递充份地执行了伦理及科学审查，详情如下：

- 申请 / 呈递类别：
  - ☑ 初始审查　　☐ 复审
  - ☐ 定期跟踪审查　　☐ 修正案审查
  - ☐ 结题审查　　☐ 新信息审查
  - ☐ 严重不良事件审查　　☐ 不依从 / 违背方案审查
  - ☐ 其他
- 审查方式：　☐ 会议审查　　☑ 简易审查
- 审查日期：　2024.10.23
- 审查委员会/委员人数情况：　委员会名称：科研项目伦理审查委员会
  委员会总人数：33 人，投票委员人数：2 人。
- 投票情况：　同意票数：2；修改后简易审查票数：0；修改后会议审查票数：0；不同意票数：0；回避票数：0；
  总计票数：2。
- 已审查的文件：　详见附件一
- 评审委员：　详见附件二

香港大学

经本会评审委员审查后，本会已就 您的申请 / 呈递做出决定，现特函通知如下：

- 审查决定：　　　　　　　　**同意**
- 意见（如适用）：
- 定期跟踪审查：　　　　　　于临床研究进行期间并自初始审查同意日起计每（☐3　☐6　☑12　☐不适用）个月提交定期跟踪审查报告

您作为该项医学研究的研究者，必须根据本伦理委员会之要求进行该研究，并需在研究期间与本会保持紧密的沟通及承担研究者的责任，包括（但不限于）：

- 留意和遵守本会的标准操作规程内的所有适用要求；
- 根据本会的标准操作规程及本批件要求的时限提交定期跟踪报告；
- 在未得本会的书面批准前，不可执行任何对研究方案或研究文件 / 材料的修改 / 变更，除非为了消除任何对受试者的紧急危害，或有关的修改 / 变更仅属于行政或后勤的性质；
- 向本会报告任何可能对受试者的权益、安全和健康或对临床研究的妥善管理产生不利影响的新信息；
- 向本会报告任何在研究期间发生并可能对受试者的权益、安全和健康产生不利影响的不依从 / 违背方案事件；
- 根据本会的标准操作规程的要求，提交在您的研究中心内发现的严重不良事件及由研究中心外通报的可疑且非预期的严重不良事件报告；
- 根据本会的标准操作规程的要求，在暂停、终止研究前提交暂停/终止临床研究审查申请表，以及完成研究后提交结题报告。

________________________（签字）

潘伟生
香港大学深圳医院科研项目伦理审查委员会
副主任委员
香港大学深圳医院　神经医学中心顾问医生

____

香港大学深圳医院医学伦理委员会盖章

附件一：

已审查的文件

港大深圳医院医学伦理委员会已审查的文件包括：
1.初始审查申请.
2.申请书.
3.项目材料诚信承诺书.
4.研究方案（版本号：V2.0，版本日期：2024年09月30日）.
5.知情同意书（版本号：V1.0，版本日期：2024年10月18日）.
6.科学性审查意见.
7.利益冲突声明.
8.研究人员的名单.
9.调查问卷表（中英文版）.
10.参考文献.

深圳医院
伦理委员会

附件二：

评审委员名单

参与审查有关申请 / 呈递并代表港大深圳医院医学伦理委员会作出审查决定的委员包括：

姓名	职业及所属单位	性别	委员类别<br>（在对应栏打“√”）			
			科学委员	非科学委员	法律专家委员	独立委员
潘伟生	神经医学中心 顾问医生<br>香港大学深圳医院	男	√			
陈佩玲	药学部 部门副主任<br>香港大学深圳医院	女	√			
